# Commentary: Parent-Reported Behavioral and Psychiatric Problems Mediate the Relationship between Sleep-Disordered Breathing and Cognitive Deficits in School-Aged Children

**DOI:** 10.3389/fneur.2017.00597

**Published:** 2017-11-13

**Authors:** Fiona Barwick, Christian Guilleminault

**Affiliations:** ^1^Division of Sleep Medicine, Department of Psychiatry and Behavioral Sciences, Stanford University School of Medicine, Stanford, United States; ^2^Neurology, Stanford University School of Medicine, Stanford, United States

**Keywords:** pediatrics, sleep disordered breathing, snoring, clinical assessment, behavioral symptoms, cognition

The association between sleep-disordered breathing (SDB) and behavior problems has been well established since 1982. Smith and colleagues attempt to expand upon this known association (1). They are to be commended for identifying an important and clinically relevant question: Why does cognitive functioning fail to improve following treatment of SDB in school-age children? However, we believe that their attempt to answer this question falls short conceptually and statistically. Their operationalization of such broad constructs as “cognitive functioning” and “behavior problems” appears to be inadequate, and their statistical modeling seems incomplete. As a result, the answer that they infer to the question that they pose—that behavior problems are the mediating factor hindering improvement in cognitive functioning after treatment of sleep disordered breathing in children—may be misconceived.

First, the authors ignore the caveat emphasized by all well-known neuropsychologists regarding the appropriate use of tests and measures in clinical assessment (2–4). As David Wechsler, Jerome Sattler, and other luminaries in the field of neuropsychology emphasize in their definitive texts on the assessment of intellectual functioning and cognitive abilities in children and adults, single test scores and even more importantly subtest scores should never be interpreted in isolation, as they yield an incomplete and unreliable picture of individual functioning, whether cognitive, behavioral, social, or psychological. The recommended approach for evaluating any functional domain is to use multiple tests to characterize the domain, examine all test scores, look for consistent and converging patterns in the data, and interpret these patterns in the context of corroborating information provided by parents, teachers, and other sources. Using individual subtest scores to characterize complex and variable functional domains leaves too much potential for error and misinterpretation.

Despite these caveats, the authors use subscale scores from only two screening measures completed by parents [Child Behavior Checklist-Revised (CBCL), Connors’ Parent Rating Scales-Revised (CPRS-R)] to characterize complex functional domains such as “problematic behaviors” and “psychiatric concerns.” Their chosen measures may be standardized, well-validated, and widely used, but they are screening measures designed to have greater sensitivity than specificity. As evidence of the appropriateness of their chosen measures, the authors cite single studies with only modest results on childhood disorders unrelated to the ones they are examining (bipolar disorder or autism spectrum disorder from citations 31 and 32) or refer to meta-analyses using scales that they do not employ in their study (CBCL-Attention Problem from citation 35). The authors also use NEPSY individual subtest scores to “capture” cognitive deficits. No matter how strong the overall psychometrics of their chosen cognitive measures, which the authors duly provide, they cannot overcome the limitations inherent in using single subtest scores to characterize entire domains of complex functioning. Because of these psychometric limitations, neither we nor the authors can be confident that the conceptual constructs they use in their analyses are adequately and validly operationalized. This is especially true when behavior and cognitive performance are highly variable and constantly changing, as is true for developing children.

Second, the authors attempt to show that behavior problems mediate between SDB and cognitive functioning. Their choice of recently developed variations on structural equation modeling that allow assessment of mediators (resampling-based mediation and ratio-of-mediator-probability-weights) indicates an understanding of quantitative methods that is likely more sophisticated than ours. It is unclear, however, why the authors test only models where behavior mediates between SDB and cognitive functioning, as cognition might equally mediate between SDB and behavioral functioning. Furthermore, the correlation between SDB and cognition or behavior is low (−0.21 or 0.26, respectively Ref. (1) from Figure 1) and likely inflated by the size of the sample (5). This suggests that SDB accounts for only limited variance in either construct, raising questions about the clinical relevance or explanatory power of the model. Finally, the authors fail to measure and include important covariates, such as child IQ, parent education level, and family socioeconomic status, which can correlate even more highly with cognitive functioning and behavior problems than the covariates that they do include (age, sex, race, BMI) (6) and which might render the impact of sleep disordered breathing on behavior problems and cognitive functioning negligible. The statistical model that they offer appears to be incomplete and not well articulated, leaving the reader unconvinced that the authors have specified the correct model for their data.

## Author Contributions

CG and FB wrote the commentary jointly.

## Conflict of Interest Statement

The authors declare that the research was conducted in the absence of any commercial or financial relationships that could be construed as a potential conflict of interest.
